# *meso*-4,4′-Dimeth­oxy-2,2′-{[(3a*R*,7a*S*)-2,3,3a,4,5,6,7,7a-octa­hydro-1*H*-benz­imidazole-1,3-di­yl]bis­(methyl­ene)}diphenol

**DOI:** 10.1107/S1600536813015092

**Published:** 2013-06-08

**Authors:** Augusto Rivera, Diego Quiroga, Jaime Ríos-Motta, Monika Kučeraková, Michal Dušek

**Affiliations:** a Universidad Nacional de Colombia, Sede Bogotá, Facultad de Ciencias, Departamento de Química, Cra 30 No. 45-03, Bogotá, Código Postal 111321, Colombia; b Institute of Physics ASCR, v.v.i., Na Slovance 2, 182 21 Praha 8, Czech Republic

## Abstract

The title compound, C_23_H_30_N_2_O_4_, a *di*-Mannich base derived from 4-meth­oxy­phenol and *cis-*1,2-di­amine­cyclo­hexane, has a perhydro­benzimidazolidine nucleus, in which the cyclo­hexane ring adopts a chair conformation and the heterocyclic ring has a half-chair conformation with a C—N—C—C torsion angles of −48.14 (15) and −14.57 (16)°. The mean plane of the heterocycle makes dihedral angles of 86.29 (6) and 78.92 (6)° with the pendant benzene rings. The mol­ecular structure of the title compound shows the presence of two inter­actions between the N atoms of the imidazolidine ring and the hydroxyl groups through intra­molecular O—H⋯N hydrogen bonds with graph-set motif *S*(6). The unobserved lone pairs of the N atoms are presumed to be disposed in a *syn* conformation, being only the second example of an exception to the typical ‘rabbit-ears’ effect in 1,2-di­amines.

## Related literature

For related structures, see: Rivera *et al.* (2011 ▶, 2013*a* ▶). For the preparation of the title compound, see: Rivera *et al.* (2013*b* ▶). For standard bond lengths, see: Allen *et al.* (1987 ▶). For hydrogen-bond graph-set nomenclature, see: Bernstein *et al.* (1995 ▶). For a discussion of the ‘rabbit-ear’ effect in 1,2-di­amines, see: Hutchins *et al.* (1968 ▶). For background to this work, see: Van den Enden & Geise (1981 ▶); Geise *et al.* (1971 ▶). For the extinction correction, see: Becker & Coppens (1974 ▶).
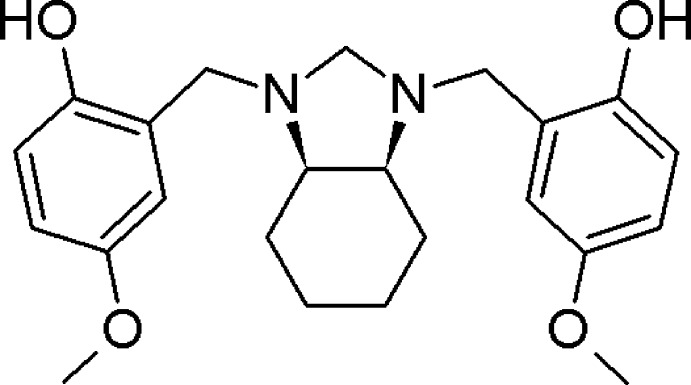


## Experimental

### 

#### Crystal data

C_23_H_30_N_2_O_4_
*M* *_r_* = 398.5
Orthorhombic, 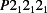

*a* = 6.4135 (3) Å
*b* = 11.4099 (6) Å
*c* = 27.8249 (14) Å
*V* = 2036.15 (18) Å^3^
*Z* = 4
Cu *K*α radiation
μ = 0.72 mm^−1^
*T* = 120 K
0.21 × 0.13 × 0.13 mm

#### Data collection

Agilent Xcalibur (Atlas, Gemini ultra) diffractometer
Absorption correction: multi-scan (*CrysAlis PRO*; Agilent, 2010 ▶) *T*_min_ = 0.341, *T*_max_ = 1
5920 measured reflections
3491 independent reflections
3103 reflections with *I* > 3σ(*I*)
*R*_int_ = 0.023

#### Refinement

*R*[*F* ^2^ > 2σ(*F* ^2^)] = 0.031
*wR*(*F* ^2^) = 0.080
*S* = 1.13
3491 reflections
269 parameters
1 restraint
H atoms treated by a mixture of independent and constrained refinement
Δρ_max_ = 0.11 e Å^−3^
Δρ_min_ = −0.09 e Å^−3^

### 

Data collection: *CrysAlis PRO* (Agilent, 2010 ▶); cell refinement: *CrysAlis PRO*; data reduction: *CrysAlis PRO*; program(s) used to solve structure: *SUPERFLIP* (Palatinus & Chapuis 2007 ▶); program(s) used to refine structure: *JANA2006* (Petříček *et al.*, 2006 ▶); molecular graphics: *DIAMOND* (Brandenburg & Putz, 2005 ▶); software used to prepare material for publication: *JANA2006*.

## Supplementary Material

Crystal structure: contains datablock(s) global, I. DOI: 10.1107/S1600536813015092/sj5324sup1.cif

Structure factors: contains datablock(s) I. DOI: 10.1107/S1600536813015092/sj5324Isup2.hkl

Additional supplementary materials:  crystallographic information; 3D view; checkCIF report

## Figures and Tables

**Table 1:** Hydrogen-bond geometry (Å, °)

*D*—H⋯*A*	*D*—H	H⋯*A*	*D*⋯*A*	*D*—H⋯*A*
O3—H1*o*3⋯N2	0.93 (2)	1.78 (2)	2.6443 (19)	154.4 (19)
O1—H1*o*1⋯N1	0.93 (2)	1.83 (2)	2.6638 (19)	148.8 (18)

**Fig. 1.**
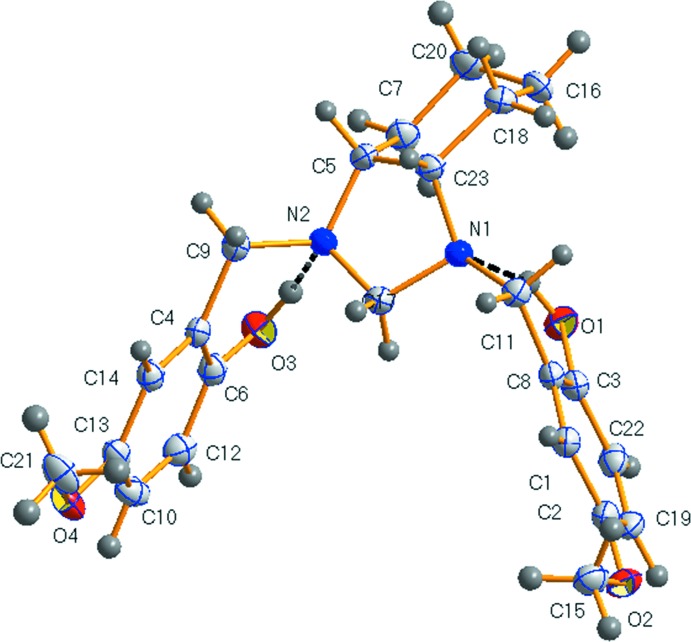
A perspective view of the title compound. Displacement ellipsoids are drawn at the 50% probability level and H atoms are shown as small spheres of arbitrary radii. Hydrogen bonds are drawn as dashed lines.

## supplementary crystallographic information

### Comment

Structural features and stereochemistry of 1,1- and 1,2-diamine functionalities play an important role in the potential reactivity of cyclic aminals. We have demonstrated that cyclic aminals are precursors of di-Mannich bases, an interesting family of 1,2-diamines, where the lone pairs usually adopt an anti conformation (Rivera, et al. 2011) to avoid electron pair repulsions (the rabbit-ears effect) (Hutchins et al. 1968). Although a syn conformation is not typical in this kind of compounds, we obtained 4,4'-difluoro-2,2'-{[(3aR,7aS)-2,3,3a,4,5,6,7,7a-octahydro-1H-1,3-benzimidazole-1,3-diyl]bis(methylene)}diphenol (Rivera et al., 2013a), one exception of the "rabbit-ears effect" (Hutchins et al., 1968). Here we report the synthesis and crystal structure of the title compound (I).

The molecular structure and atom-numbering scheme for (I) are shown in Fig. 1. The bond lengths are close to normal (Allen et al., 1987). The crystal structure of (I) shows two intramolecular hydrogen bonds with graph-set motif S(6) (Bernstein et al., 1995) (Table 1), where the N···H distances and the N···O distances are shorter (by about 0.06 Å and 0.03 Å, respectively) than the observed values in a related structure (Rivera, et al. 2013a). These results suggest that the electronic character of the< i>para substituent in the aromatic rings does not significantly influence the strength of the intermolecular hydrogen bonds in these compounds.

The cyclohexane ring adopts a chair conformation where the endocyclic C—C—C bond angles are distorted from the normal tetrahedral bond angles in a chair conformation (Geise et al., 1971), since these values are in the range of 110.29 (15)° to 114.50 (14)°. The imidazolidine ring adopts a half chair conformation (Van den Enden & Geise, 1981), where the nitrogen lone pairs are oriented in a syn disposition and the benzyl groups are located in 1,3-diequatorial positions.

The dihedral angle between the aromatic rings is 49.19 (52) °. The C3—C8 and C6—C4 bonds are the longest and the C10—C12 and C19—C22 bond are the shortest in the aromatic rings.

### Experimental

A solution of *p*-methoxyphenol (2.00 mmol) in dioxane (3 ml) was added dropwise to a stirred solution of (2*S*,7*R*,11*S*,16*R*)-1,8,10,17-tetraazapentacyclo[8.8.1.1.^8,17^0.^2,7^0^11,16^]icosane (276 mg, 1.00 mmol) in dioxane (3 ml). The mixture was stirred for 15 min at room temperature and then water (4 ml) was added. The mixture was heated at 313 K during 30 h. After cooling to room temperature, the solvent was removed *in vacuo* and the crude product was purified by chromatography on a silica column and subjected to gradient elution with light petroleum ether: ethyl acetate (yield 45%, *M*.p. = 405–406 K). Single crystals of (I) were grown from a CHCl_3_ solution by slow evaporation of the solvent at room temperature over a period of about 2 weeks.

### Refinement

The hydroxyl hydrogen atoms were found in difference Fourier maps and their coordinates were refined with a distance restraint d(O—H) = 0.926 Å with σ 0.01. All other H atoms atoms were kept in the geometrically correct positions with C—H distance 0.96 A. The isotropic atomic displacement parameters of hydrogen atoms were evaluated as 1.2×*U*_eq_ of the parent atom.

### Crystal data

**Table:**

C_23_H_30_N_2_O_4_	*F*(000) = 856
*M**_r_* = 398.5	*D*_x_ = 1.300 Mg m^−^^3^
Orthorhombic, *P*2_1_2_1_2_1_	Cu *K*α radiation, λ = 1.5418 Å
Hall symbol: P 2ac 2ab	Cell parameters from 3881 reflections
*a* = 6.4135 (3) Å	θ = 4.2–67.0°
*b* = 11.4099 (6) Å	µ = 0.72 mm^−^^1^
*c* = 27.8249 (14) Å	*T* = 120 K
*V* = 2036.15 (18) Å^3^	Polygon shape, white
*Z* = 4	0.21 × 0.13 × 0.13 mm

### Data collection

**Table:**

Agilent Xcalibur (Atlas, Gemini ultra) diffractometer	3491 independent reflections
Radiation source: Enhance Ultra (Cu) X-ray Source	3103 reflections with *I* > 3σ(*I*)
Mirror monochromator	*R*_int_ = 0.023
Detector resolution: 10.3784 pixels mm^-1^	θ_max_ = 67.1°, θ_min_ = 3.2°
ω scans	*h* = −7→4
Absorption correction: multi-scan (*CrysAlis PRO*; Agilent, 2010)	*k* = −13→12
*T*_min_ = 0.341, *T*_max_ = 1	*l* = −30→32
5920 measured reflections	

### Refinement

**Table:**

Refinement on *F*^2^	H atoms treated by a mixture of independent and constrained refinement
*R*[*F*^2^ > 2σ(*F*^2^)] = 0.031	Weighting scheme based on measured s.u.'s *w* = 1/(σ^2^(*I*) + 0.0016*I*^2^)
*wR*(*F*^2^) = 0.080	(Δ/σ)_max_ = 0.014
*S* = 1.13	Δρ_max_ = 0.11 e Å^−^^3^
3491 reflections	Δρ_min_ = −0.09 e Å^−^^3^
269 parameters	Extinction correction: B-C type 1 Gaussian isotropic (Becker & Coppens, 1974)
1 restraint	Extinction coefficient: 1800 (300)
114 constraints	

### Special details

**Table:**

Refinement. The refinement was carried out against all reflections. The conventional *R*-factor is always based on *F*. The goodness of fit as well as the weighted *R*-factor are based on *F* and *F*^2^ for refinement carried out on *F* and *F*^2^, respectively. The threshold expression is used only for calculating *R*-factors *etc*. and it is not relevant to the choice of reflections for refinement.The program used for refinement, Jana2006, uses the weighting scheme based on the experimental expectations, see _refine_ls_weighting_details, that does not force *S* to be one. Therefore the values of *S* are usually larger than the ones from the *SHELX* program.

### Fractional atomic coordinates and isotropic or equivalent isotropic displacement parameters (Å^2^)

**Table:**

	*x*	*y*	*z*	*U*_iso_*/*U*_eq_	
O1	0.9143 (2)	0.54863 (11)	0.19640 (4)	0.0301 (4)	
O2	0.5296 (2)	0.11931 (11)	0.22143 (5)	0.0331 (4)	
O3	1.1573 (2)	0.56734 (11)	0.02995 (4)	0.0307 (4)	
O4	0.75546 (19)	0.25342 (11)	−0.08969 (5)	0.0323 (4)	
N1	0.6253 (2)	0.60135 (11)	0.13114 (5)	0.0214 (4)	
N2	0.7838 (2)	0.63852 (12)	0.05764 (5)	0.0236 (4)	
C1	0.5156 (3)	0.31779 (14)	0.18880 (5)	0.0235 (5)	
C2	0.6158 (3)	0.22830 (15)	0.21334 (6)	0.0263 (5)	
C3	0.8117 (3)	0.44415 (15)	0.20127 (6)	0.0239 (5)	
C4	0.8426 (3)	0.51409 (14)	−0.01265 (5)	0.0227 (4)	
C5	0.7086 (3)	0.74987 (14)	0.07934 (6)	0.0240 (5)	
C6	1.0495 (3)	0.49232 (15)	0.00056 (6)	0.0249 (5)	
C7	0.8898 (3)	0.80893 (16)	0.10624 (6)	0.0291 (5)	
C8	0.6101 (3)	0.42686 (14)	0.18311 (5)	0.0225 (5)	
C9	0.7333 (3)	0.62223 (15)	0.00643 (6)	0.0241 (5)	
C10	1.0459 (3)	0.31475 (16)	−0.04591 (6)	0.0299 (5)	
C11	0.4897 (3)	0.52615 (15)	0.16028 (6)	0.0232 (5)	
C12	1.1484 (3)	0.39175 (15)	−0.01597 (6)	0.0285 (5)	
C13	0.8412 (3)	0.33593 (14)	−0.05968 (6)	0.0253 (5)	
C14	0.7399 (3)	0.43469 (14)	−0.04254 (6)	0.0234 (5)	
C15	0.3383 (3)	0.09474 (15)	0.19795 (7)	0.0318 (5)	
C16	0.6655 (3)	0.84873 (15)	0.17768 (6)	0.0296 (5)	
C17	0.6964 (3)	0.54281 (14)	0.08724 (6)	0.0246 (5)	
C18	0.4760 (3)	0.79993 (15)	0.15171 (6)	0.0267 (5)	
C19	0.8144 (3)	0.24615 (15)	0.23194 (6)	0.0277 (5)	
C20	0.8172 (3)	0.90189 (16)	0.14189 (7)	0.0339 (6)	
C21	0.5386 (3)	0.26108 (18)	−0.09885 (7)	0.0377 (6)	
C22	0.9116 (3)	0.35333 (16)	0.22549 (6)	0.0273 (5)	
C23	0.5338 (3)	0.71070 (14)	0.11279 (6)	0.0230 (5)	
H1c1	0.379468	0.30444	0.17556	0.0282*	
H1c5	0.65943	0.806783	0.0566	0.0288*	
H1c7	0.969757	0.750452	0.122842	0.0349*	
H2c7	0.983602	0.843718	0.083447	0.0349*	
H1c9	0.777998	0.689708	−0.011408	0.0289*	
H2c9	0.585279	0.613214	0.002786	0.0289*	
H1c10	1.11646	0.245973	−0.057325	0.0359*	
H1c11	0.380664	0.494706	0.140473	0.0278*	
H2c11	0.424373	0.572215	0.184908	0.0278*	
H1c12	1.289471	0.375787	−0.006445	0.0342*	
H1c14	0.597307	0.448657	−0.051337	0.028*	
H1c15	0.291175	0.017919	0.206897	0.0381*	
H2c15	0.235901	0.151753	0.207273	0.0381*	
H3c15	0.358284	0.097924	0.163784	0.0381*	
H1c16	0.621918	0.907719	0.200132	0.0355*	
H2c16	0.733433	0.786893	0.195069	0.0355*	
H1c17	0.579091	0.508786	0.070973	0.0295*	
H2c17	0.804762	0.487985	0.095061	0.0295*	
H1c18	0.398726	0.863134	0.137522	0.0321*	
H2c18	0.383947	0.763989	0.174577	0.0321*	
H1c19	0.883769	0.184533	0.249162	0.0333*	
H1c20	0.935569	0.932931	0.158751	0.0407*	
H2c20	0.749972	0.964503	0.124838	0.0407*	
H1c21	0.49724	0.198114	−0.119656	0.0453*	
H2c21	0.463462	0.255624	−0.069084	0.0453*	
H3c21	0.50801	0.334683	−0.114005	0.0453*	
H1c22	1.049675	0.365199	0.237874	0.0327*	
H1c23	0.400514	0.701537	0.097533	0.0276*	
H1o3	1.050 (3)	0.6060 (18)	0.0456 (7)	0.0369*	
H1o1	0.834 (3)	0.5936 (17)	0.1758 (7)	0.0361*	

### Atomic displacement parameters (Å^2^)

**Table:**

	*U*^11^	*U*^22^	*U*^33^	*U*^12^	*U*^13^	*U*^23^
O1	0.0312 (7)	0.0281 (7)	0.0309 (6)	−0.0030 (6)	−0.0068 (5)	0.0021 (5)
O2	0.0438 (7)	0.0216 (6)	0.0340 (6)	0.0000 (6)	−0.0026 (6)	0.0074 (5)
O3	0.0261 (6)	0.0333 (7)	0.0328 (6)	−0.0028 (6)	−0.0018 (5)	−0.0048 (5)
O4	0.0330 (7)	0.0310 (7)	0.0330 (6)	−0.0036 (6)	0.0062 (5)	−0.0123 (5)
N1	0.0260 (7)	0.0180 (6)	0.0202 (6)	0.0011 (6)	0.0004 (5)	−0.0003 (5)
N2	0.0321 (8)	0.0189 (7)	0.0197 (6)	0.0012 (6)	−0.0010 (6)	−0.0011 (5)
C1	0.0268 (9)	0.0236 (9)	0.0202 (7)	0.0013 (7)	0.0010 (7)	0.0005 (6)
C2	0.0346 (10)	0.0240 (9)	0.0204 (7)	0.0042 (8)	0.0044 (7)	0.0022 (6)
C3	0.0284 (9)	0.0239 (8)	0.0194 (7)	0.0018 (7)	−0.0002 (6)	−0.0010 (6)
C4	0.0281 (9)	0.0227 (8)	0.0174 (7)	0.0002 (8)	0.0035 (7)	0.0020 (6)
C5	0.0348 (9)	0.0177 (8)	0.0195 (7)	0.0026 (8)	−0.0015 (7)	0.0017 (6)
C6	0.0268 (8)	0.0263 (9)	0.0217 (7)	−0.0038 (7)	0.0019 (7)	0.0006 (6)
C7	0.0325 (10)	0.0258 (9)	0.0289 (8)	−0.0052 (8)	0.0034 (7)	−0.0014 (7)
C8	0.0269 (9)	0.0240 (8)	0.0165 (7)	0.0029 (7)	0.0011 (6)	−0.0014 (6)
C9	0.0283 (9)	0.0239 (8)	0.0201 (7)	0.0014 (7)	−0.0007 (6)	0.0011 (6)
C10	0.0302 (9)	0.0242 (9)	0.0354 (9)	0.0007 (8)	0.0105 (8)	−0.0022 (7)
C11	0.0240 (8)	0.0235 (8)	0.0220 (7)	0.0001 (7)	−0.0007 (6)	0.0015 (6)
C12	0.0255 (9)	0.0282 (9)	0.0319 (8)	0.0009 (8)	0.0032 (7)	0.0037 (7)
C13	0.0300 (9)	0.0223 (8)	0.0237 (8)	−0.0048 (7)	0.0062 (7)	−0.0014 (6)
C14	0.0242 (8)	0.0253 (8)	0.0206 (7)	−0.0013 (7)	0.0026 (6)	0.0005 (6)
C15	0.0360 (10)	0.0245 (9)	0.0349 (9)	−0.0012 (8)	0.0039 (8)	0.0040 (7)
C16	0.0400 (10)	0.0239 (8)	0.0249 (8)	0.0025 (8)	−0.0039 (8)	−0.0069 (6)
C17	0.0313 (9)	0.0208 (8)	0.0217 (7)	0.0035 (7)	0.0017 (7)	−0.0003 (6)
C18	0.0305 (9)	0.0222 (8)	0.0274 (8)	0.0053 (8)	0.0010 (7)	−0.0010 (7)
C19	0.0334 (10)	0.0275 (9)	0.0222 (7)	0.0091 (8)	0.0003 (7)	0.0035 (7)
C20	0.0425 (11)	0.0244 (9)	0.0348 (9)	−0.0045 (9)	−0.0052 (8)	−0.0047 (7)
C21	0.0345 (10)	0.0397 (11)	0.0389 (10)	−0.0068 (9)	0.0015 (8)	−0.0152 (8)
C22	0.0255 (9)	0.0341 (9)	0.0223 (8)	0.0052 (8)	−0.0024 (7)	0.0008 (7)
C23	0.0248 (9)	0.0202 (8)	0.0239 (7)	0.0020 (7)	−0.0033 (7)	0.0002 (6)

### Geometric parameters (Å, º)

**Table:**

O1—C3	1.368 (2)	C9—H1c9	0.96
O1—H1o1	0.93 (2)	C9—H2c9	0.96
O2—C2	1.380 (2)	C10—C12	1.378 (3)
O2—C15	1.418 (2)	C10—C13	1.389 (3)
O3—C6	1.371 (2)	C10—H1c10	0.96
O3—H1o3	0.93 (2)	C11—H1c11	0.96
O4—C13	1.373 (2)	C11—H2c11	0.96
O4—C21	1.417 (2)	C12—H1c12	0.96
N1—C11	1.466 (2)	C13—C14	1.385 (2)
N1—C17	1.465 (2)	C14—H1c14	0.96
N1—C23	1.470 (2)	C15—H1c15	0.96
N2—C5	1.487 (2)	C15—H2c15	0.96
N2—C9	1.473 (2)	C15—H3c15	0.96
N2—C17	1.478 (2)	C16—C18	1.520 (3)
C1—C2	1.386 (2)	C16—C20	1.519 (3)
C1—C8	1.393 (2)	C16—H1c16	0.96
C1—H1c1	0.96	C16—H2c16	0.96
C2—C19	1.390 (3)	C17—H1c17	0.96
C3—C8	1.402 (2)	C17—H2c17	0.96
C3—C22	1.392 (2)	C18—C23	1.532 (2)
C4—C6	1.399 (3)	C18—H1c18	0.96
C4—C9	1.515 (2)	C18—H2c18	0.96
C4—C14	1.395 (2)	C19—C22	1.384 (3)
C5—C7	1.538 (2)	C19—H1c19	0.96
C5—C23	1.524 (2)	C20—H1c20	0.96
C5—H1c5	0.96	C20—H2c20	0.96
C6—C12	1.390 (2)	C21—H1c21	0.96
C7—C20	1.525 (3)	C21—H2c21	0.96
C7—H1c7	0.96	C21—H3c21	0.96
C7—H2c7	0.96	C22—H1c22	0.96
C8—C11	1.511 (2)	C23—H1c23	0.96
			
C3—O1—H1o1	106.0 (13)	C6—C12—H1c12	119.71
C2—O2—C15	116.72 (13)	C10—C12—H1c12	119.71
C6—O3—H1o3	101.6 (13)	O4—C13—C10	115.28 (15)
C13—O4—C21	117.40 (14)	O4—C13—C14	125.39 (16)
C11—N1—C17	112.27 (12)	C10—C13—C14	119.32 (16)
C11—N1—C23	116.87 (13)	C4—C14—C13	120.79 (16)
C17—N1—C23	102.81 (12)	C4—C14—H1c14	119.6
C5—N2—C9	115.42 (13)	C13—C14—H1c14	119.6
C5—N2—C17	106.39 (12)	O2—C15—H1c15	109.47
C9—N2—C17	111.24 (13)	O2—C15—H2c15	109.47
C2—C1—C8	120.84 (16)	O2—C15—H3c15	109.47
C2—C1—H1c1	119.58	H1c15—C15—H2c15	109.47
C8—C1—H1c1	119.58	H1c15—C15—H3c15	109.47
O2—C2—C1	123.98 (16)	H2c15—C15—H3c15	109.47
O2—C2—C19	116.01 (15)	C18—C16—C20	110.30 (14)
C1—C2—C19	120.01 (16)	C18—C16—H1c16	109.47
O1—C3—C8	122.03 (15)	C18—C16—H2c16	109.47
O1—C3—C22	118.37 (15)	C20—C16—H1c16	109.47
C8—C3—C22	119.60 (15)	C20—C16—H2c16	109.47
C6—C4—C9	119.44 (14)	H1c16—C16—H2c16	108.63
C6—C4—C14	119.29 (15)	N1—C17—N2	104.22 (12)
C9—C4—C14	121.26 (15)	N1—C17—H1c17	109.47
N2—C5—C7	109.08 (14)	N1—C17—H2c17	109.47
N2—C5—C23	103.65 (13)	N2—C17—H1c17	109.47
N2—C5—H1c5	114.64	N2—C17—H2c17	109.47
C7—C5—C23	112.79 (13)	H1c17—C17—H2c17	114.25
C7—C5—H1c5	105.82	C16—C18—C23	112.72 (15)
C23—C5—H1c5	111.05	C16—C18—H1c18	109.47
O3—C6—C4	121.62 (15)	C16—C18—H2c18	109.47
O3—C6—C12	118.85 (16)	C23—C18—H1c18	109.47
C4—C6—C12	119.52 (16)	C23—C18—H2c18	109.47
C5—C7—C20	113.00 (15)	H1c18—C18—H2c18	106.02
C5—C7—H1c7	109.47	C2—C19—C22	119.58 (16)
C5—C7—H2c7	109.47	C2—C19—H1c19	120.21
C20—C7—H1c7	109.47	C22—C19—H1c19	120.21
C20—C7—H2c7	109.47	C7—C20—C16	110.14 (15)
H1c7—C7—H2c7	105.7	C7—C20—H1c20	109.47
C1—C8—C3	119.06 (15)	C7—C20—H2c20	109.47
C1—C8—C11	119.71 (15)	C16—C20—H1c20	109.47
C3—C8—C11	121.14 (15)	C16—C20—H2c20	109.47
N2—C9—C4	109.87 (13)	H1c20—C20—H2c20	108.8
N2—C9—H1c9	109.47	O4—C21—H1c21	109.47
N2—C9—H2c9	109.47	O4—C21—H2c21	109.47
C4—C9—H1c9	109.47	O4—C21—H3c21	109.47
C4—C9—H2c9	109.47	H1c21—C21—H2c21	109.47
H1c9—C9—H2c9	109.07	H1c21—C21—H3c21	109.47
C12—C10—C13	120.47 (16)	H2c21—C21—H3c21	109.47
C12—C10—H1c10	119.76	C3—C22—C19	120.89 (16)
C13—C10—H1c10	119.76	C3—C22—H1c22	119.56
N1—C11—C8	111.62 (14)	C19—C22—H1c22	119.56
N1—C11—H1c11	109.47	N1—C23—C5	99.62 (13)
N1—C11—H2c11	109.47	N1—C23—C18	114.52 (13)
C8—C11—H1c11	109.47	N1—C23—H1c23	114.61
C8—C11—H2c11	109.47	C5—C23—C18	114.50 (14)
H1c11—C11—H2c11	107.24	C5—C23—H1c23	114.63
C6—C12—C10	120.58 (17)	C18—C23—H1c23	99.77

### Hydrogen-bond geometry (Å, º)

**Table:**

*D*—H···*A*	*D*—H	H···*A*	*D*···*A*	*D*—H···*A*
O3—H1*o*3···N2	0.93 (2)	1.78 (2)	2.6443 (19)	154.4 (19)
O1—H1*o*1···N1	0.93 (2)	1.83 (2)	2.6638 (19)	148.8 (18)
